# The importance of side branches in modeling 3D hemodynamics from angiograms for patients with coronary artery disease

**DOI:** 10.1038/s41598-019-45342-5

**Published:** 2019-06-20

**Authors:** Madhurima Vardhan, John Gounley, S. James Chen, Andrew M. Kahn, Jane A. Leopold, Amanda Randles

**Affiliations:** 1 Department of Biomedical Engineering, Duke University, Durham, 27708 USA; 2 Department of Medicine/Cardiology, University of Colorado AMC, Aurora, 80045 USA; 3 Division of Cardiovascular Medicine, University of California San Diego, San Diego, 92103 USA; 4 Division of Cardiovascular Medicine, Brigham and Women’s Hospital, Boston, 02115 USA

**Keywords:** Biomedical engineering, Computational science, Interventional cardiology

## Abstract

Genesis of atherosclerotic lesions in the human arterial system is critically influenced by the fluid mechanics. Applying computational fluid dynamic tools based on accurate coronary physiology derived from conventional biplane angiogram data may be useful in guiding percutaneous coronary interventions. The primary objective of this study is to build and validate a computational framework for accurate personalized 3-dimensional hemodynamic simulation across the complete coronary arterial tree and demonstrate the influence of side branches on coronary hemodynamics by comparing shear stress between coronary models with and without these included. The proposed novel computational framework based on biplane angiography enables significant arterial circulation analysis. This study shows that models that take into account flow through all side branches are required for precise computation of shear stress and pressure gradient whereas models that have only a subset of side branches are inadequate for biomechanical studies as they may overestimate volumetric outflow and shear stress. This study extends the ongoing computational efforts and demonstrates that models based on accurate coronary physiology can improve overall fidelity of biomechanical studies to compute hemodynamic risk-factors.

## Introduction

Coronary artery disease is associated with a high rate of morbidity and mortality and is recognized as the leading cause of death worldwide. Symptomatic patients may undergo diagnostic testing with a noninvasive coronary computed tomography angiogram (CTA) or invasive coronary angiography to identify obstructive lesions that narrow the vessel lumen and limit blood flow. Fluid mechanics play a critical role in determining the progression and the functional significance of stenoses in the human arterial system and coronary artery disease in particular. Clinical and experimental studies have investigated endothelial shear stress [ESS], the frictional force acting on the endothelium, and identified low ESS as a pro-atherogenic risk factor^1–9^. Low fluid velocity and the resulting low ESS on the vessel wall have been reported to be directly related to vessel wall thickening and plaque development. The functional regulation of the endothelium by local ESS has laid the foundation for computational fluid dynamic (CFD) studies as a platform to understand how mitigation of hemodynamic disruption can reduce the risk of coronary artery disease progression and potentially translate into a guide for therapeutic interventions.

There has been a recent increase in interest in using CFD to perform diagnostic wire free analyses to examine the functional significance of a coronary artery luminal stenosis. Early CFD studies used CT angiography (CTA) data to create vascular geometries because it provided isotropic 3-dimensional (3D) anatomic data and the necessary functional information in a single radiologic scan^10,11^. However, owing to the limited number of patients that undergo CTA and the fact that >1 million coronary angiography procedures are performed annually in the United States, and the high spatial (150–200 mm) and temporal (10 ms) resolution of angiography, 3D anatomic models based on coronary angiograms are now favored^12–17^. These types of state-of-the-art CFD models enable realistic modeling of multivessel coronary artery disease and calculation of coronary flow and pressure from clinically obtained medical images^18,19^.

An important determinant of the accuracy of the data derived from CFD simulations is how well the 3D model geometry approximates the anatomy as defined by the medical imaging data. The majority of existing CFD studies based on routine single plane or biplane angiographic data have analyzed only focal regions of the coronary geometry and not incorporated the effect of key anatomic features, such as side branches, in the assessment of ESS^20–23^. Prior studies done using CTA-derived geometries revealed the importance of side branches; exclusion of side branches had low sensitivity for detecting low or high ESS in the coronary vessels. Further, when 3D coronary geometries were created using intravascular optical coherence tomography fused with angiographic images to aid in the inclusion of more side branches, it was found that single vessel conduit models yielded inaccurate results. This study, however, used models with truncated side branches to model the coronaries, which likely resulted in inaccurate ESS measurements as well^20^.

The current study addresses these limitations by constructing 3D geometries of left and right coronary arterial trees with all identifiable (>1 mm) main and side branch vessels from 2D coronary angiography data. These geometries allow us to test our hypothesis that completeness of the coronary 3D geometry with inclusion of the entire side branch affects complex hemodynamics in the arterial system. We validate our methodology with patient matched coronary CTA-derived reconstructions and *in vitro* experiments. We further demonstrate the influence of the complete coronary arterial tree on ESS and intravascular pressure gradients and perform a point-to-point comparison with corresponding tree models from coronary CTAs.

## Methods

### Study population

This study does not involve any experiments on humans and/or the use of human tissue samples. This study was approved by the Partners Institutional Review Board (IRB Protocol #2015P001084). It was performed in accordance with relevant guidelines and regulations as per the approved IRB protocol. The study uses imaging datasets that were de-identified and anonymized. The protocol was reviewed by the Partners IRB and determined to not require informed consent because it was use of de-identified images/data only. Imaging datasets were acquired from 20 patients who underwent both clinically indicated coronary angiography and coronary CTA within one month at Brigham and Women’s Hospital. Clinical variables, including blood pressure, heart rate, and hematocrit at the time of coronary angiography were collected. Coronary angiography included a minimum of 4 standard orthogonal views of the left coronary circulation and 2 standard orthogonal views of the right coronary circulation. Coronary CTA images were obtained with a 64-slice scanner. All collected data were de-identified and anonymized prior to model reconstruction and simulations.

### Model reconstruction and validation

**Figure 1:**
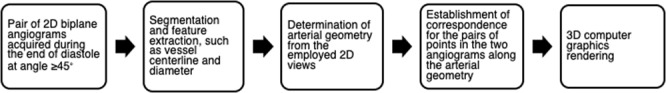
A flow diagram to describe the employed 3D reconstruction algorithm used to reconstruct a 3D arterial geometry from two biplane angiograms.

For angiogram reconstructions, we applied a 3D reconstruction algorithm described^24^. Briefly, the method uses a pair of 2D angiograms and parameters of the C-arm gantry system (e.g., separation angles > 45°, distance between X-ray spot and image intensifier, and pixel size) to create the 3D coronary skeleton by detecting vessel centerlines and cross-sectional diameter. Reconstructions were obtained at end-diastole (Fig. 1). The coronary CTA models were reconstructed by segmenting high resolution axial images (500 mm slice thickness) at 75% cardiac cycle using Mimics (Materialise, Leuven, Belgium). The 3D reconstruction models for each of the imaging modalities were validated by two cardiologists (A.K., J.L.) who were blinded to the corresponding angiogram or coronary CTA. Reconstruction of the angiograms resulted in two types of models:

- Complete coronary model (CCM): the 3D reconstruction algorithm enables the generation of a complete arterial tree comprising all identifiable vessels and side branches (>1 mm) with anatomical vessel tapering.
- Matched coronary model (MCM): CCMs reconstructed from angiogram data were pruned to match the respective CTA models with respect to the vessel type, number and length. The resulting models are referred to as matched coronary models (MCMs).

All geometric models were exported as mesh files in standard stereolithography file format for further geometric and CFD analysis. The topological and anatomical validity of the angiogram models was compared to the corresponding CTA models by calculating the Hausdorff distance (HD) between these models. Surface meshes derived from the 3D models of CTA and coronary angiogram (CA) data were aligned using N-point registration in 3-Matic (Materialise, Leuven, Belgium), by identifying discernible features such as branching patterns.

### *In vitro* geometric validation

A 3D phantom was created using the mesh file from the 2D angiogram reconstruction. The phantom was injected with Visipaque 320 ionic contrast dye and imaged in the Brigham and Women’s Hospital cardiac catheterization laboratory using standard right and left coronary artery angiographic views. A 0.014 inch coronary wire was included with each image to provide scale. The resulting angiogram from the 3D phantom was compared with the original angiogram from the patient to confirm the geometrical reconstruction. Cartesian distance was calculated to quantitatively compare the resulting angiogram along 10 locations within the arterial tree.

### Analysis of hemodynamics

CFD simulations were performed using HARVEY, a parallel hemodynamic software^25,26^. HARVEY is based on the lattice Boltzmann method (LBM), an alternate approach to solving the Navier-Stokes equations governing fluid flow. To model the coronary circulation, we simulated blood as an incompressible Newtonian fluid with a dynamic viscosity of 4 cP and density of 1060 kg/m^3^. The blood vessels are modeled as rigid walls with a no-slip boundary condition. At the outlets, a lumped parameter model was prescribed using microcirculation resistance. The coronary arterial microcirculation resistance for all geometries was computed based on the vessel diameter, mean flow and mean aortic pressure. For the inlet boundary condition, we imposed a Poiseuille profile with transient flow using velocity waveform from the literature. Transient (or pulsatile) simulations are more realistic than steady simulations as they capture the periodically changing velocity during the cardiac cycle. Pulsatile ESS derived from transient simulations is thus varying in magnitude, unidirectional and averaged over the period of the cardiac cycle.1 A convergence study was performed to obtain mesh-independent solutions, the results of which indicate that simulations were convergent at 50 mm resolution, with approximately 1.9 × 10^7^ and 1.6 × 10^7^ fluid points for CCM and MCM models respectively. Due to the large fluid domain, simulations were conducted on 1024 cores of Intel Xeon E5-2699 processors with 56 Gb/s Infiniband interconnect on the Duke Compute Cluster.

A total of 54 CFD simulations were performed for all the CCMs and MCMs with same boundary condition at the inlet. A vessel centerline was obtained using Mimics. To analyze the ESS trend in the arterial models, we computed circumferential averaged ESS at each point on the centerline. The local differences in ESS were assessed by performing a point-to-point comparison between the CCM and MCM models from all patient-specific geometry sets. Each geometry is divided in sections at a distance of 0.3 mm along the centerline, resulting in approximately 2,000 sections per geometry. Therefore, there were 17,757 sections common to 54 CCM/MCM models. CFD results are presented as 3D and 2D ESS maps. Volumetric flow rate was computed at the end of each vessel.

### Statistics

We test the normal distribution of the data with the Kolmogorov-Smirnov test. Continuous variables are reported as the mean (+/−) standard error mean (SEM) and discrete variables as counts or percentages. Continuous variables were compared with a two-tailed Student t test. Group differences were assessed with non-parametric Kruskal-Wallis or Friedman test. A value of p < 0.05 was considered significant.

## Results

### 3D coronary artery geometric reconstructions from coronary angiograms

**Figure 2:**
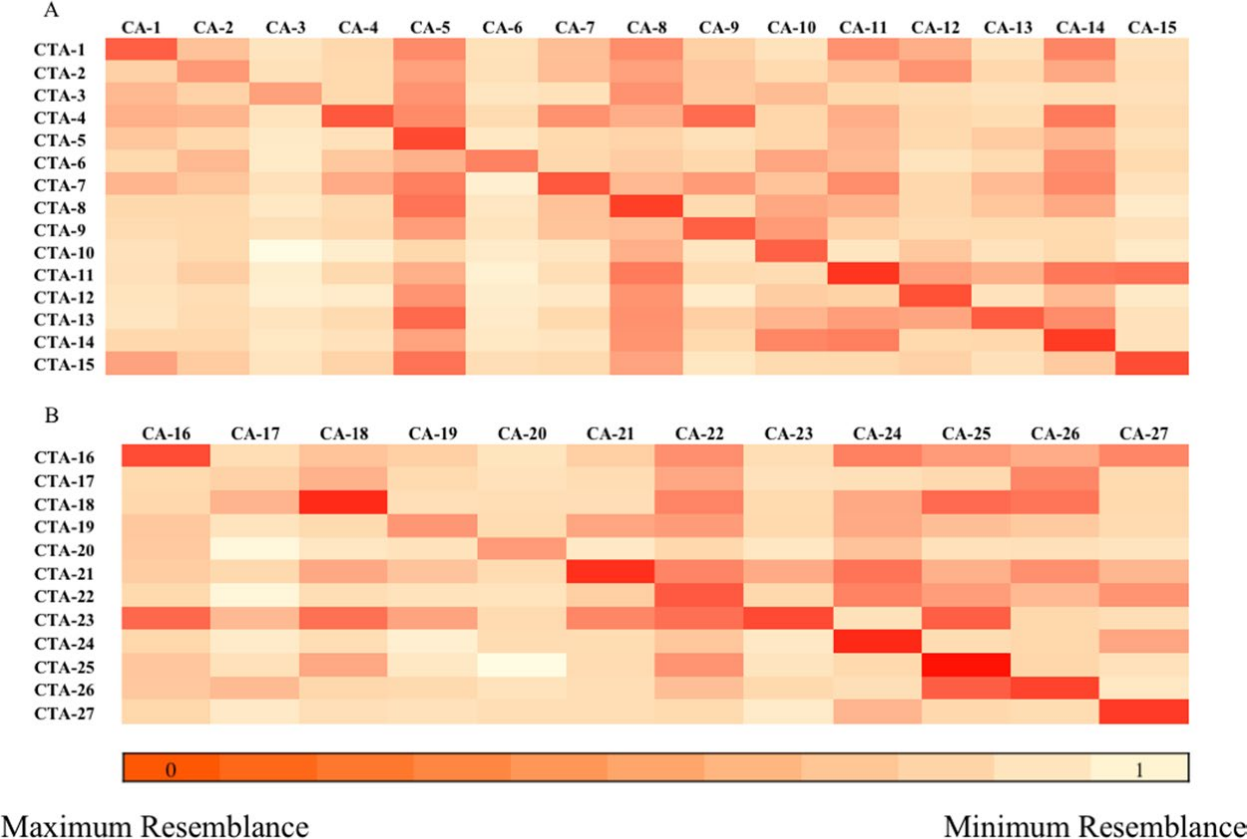
Quantitatively compared geometric topology between CTA and angiogram derived arterial models. Hausdorff distance (HD) is computed between coronary angiogram (CA) and coronary CT angiogram (CTA) derived geometries for all 27 patient cases. (**A**) Shows the heat map of the HD for the left coronary geometries. (**B**) Shows the heat map of the HD for the right coronary geometries. Highest geometric resemblances are marked by the darkest region in the heat map. The dark region along the diagonal represents correctly identified CA and CTA models from same patient.

In order to determine if the 3D coronary artery geometries derived from coronary angiograms are accurate, we utilized imaging datasets from 20 patients who underwent coronary CTA and coronary angiography testing within one month and were found to have a stenosis in at least one vessel to reconstruct a total of 15 left coronary arteries and 12 right coronary arteries from both imaging modalities. The topological accuracy of the coronary angiogram reconstructions was evaluated by aligning them with the geometries derived from the coronary CTAs, which are considered the gold-standard for 3D reconstructions for CFD of the coronary arteries and computing the HD between these reconstructions. This revealed a HD of 2.8 ± 1.7 mm for the left coronary arteries and 3.5 ± 2.2 mm for the right coronary arteries, indicating good agreement between the coronary angiogram and the CTA-derived reconstructions. To ensure that this relationship existed only between comparisons of the coronary angiogram and CTA models from the same patient, we calculated symmetric HDs for each angiogram- and CTA-derived reconstruction on a per patient basis. This also demonstrated good agreement between the matched patient CTA and angiogram-derived reconstructions as compared to unmatched patient geometries for the left coronary circulation (2.8 ± 1.7 vs. 10.6 ± 4.6 mm, p < 0.0001) and for the right coronary artery (3.5 ± 2.2 vs. 9.0 ± 3.0 mm, p < 0.0001). As anticipated, the minimum symmetric HD distance for a left or right coronary angiogram model was observed for the corresponding CTA model from the same patient (Fig. 2A,B). The high topological similarity between the reconstructions from the coronary angiograms and those from the coronary CTAs demonstrates that the complex geometry of the branching coronary tree can be recreated accurately in 3D models based on 2D coronary angiograms.

**Figure 3:**
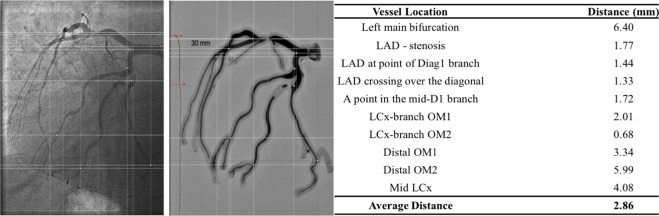
Experimental validation using 3D phantom angiography. The 3D printed phantom is obtained from the mesh file of the angiogram reconstruction. Red arrow in the phantom angiogram mark the coronary wire location and provide the scale. White cross lines identify the locations that are used for computing Cartesian distance between the angiograms.

To further validate our coronary angiogram-derived 3D geometries, we used one geometry to 3D print a phantom model and performed angiography of the phantom in the cardiac catheterization laboratory using the same views as the originally acquired patient coronary angiogram. We then compared the original patient angiograms to the angiograms obtained from the 3D phantom model at 10 locations along the arterial tree based on discernible features such as bifurcation points or a luminal stenosis (Fig. 3A). Cartesian distance, which was computed for each location, demonstrated an average distance of 2.86 mm (range 1.72–6.40 mm) between the 3D phantom and patient angiograms (Fig. 3B). This distance demonstrates the accuracy of similarity between the physical phantom model and mesh model and is lower than previously reported results^24^. Therefore, the phantom angiogram closely approximates the original patient angiogram providing additional evidence that the 3D angiogram-derived geometries are highly representative of the original patient angiograms.

### Influence of side branches on hemodynamics

**Figure 4:**
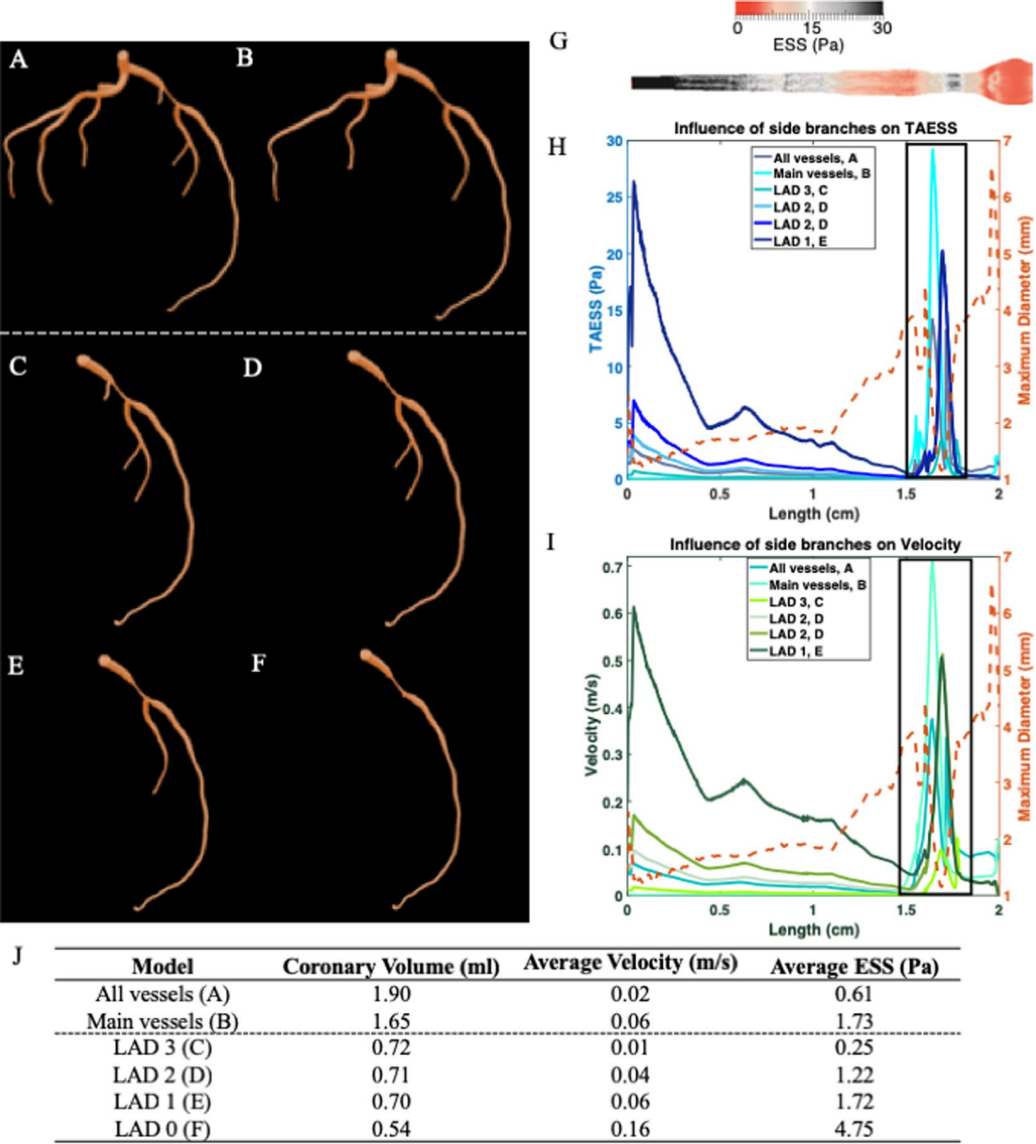
Influence of side branches on hemodynamics. (**A**) Patient 1 left coronary artery (LCA) model with all vessels. (**B**) LCA model with only main vessels (LM, LAD and LCx with major bifurcations, Diag 1 and OM1). Inlet velocity of 25 cm/sec (average diastole) is applied at the inlet for models A and B. (**C**–**F**) LAD derived models with 3, 2, 1 and 0 number of side branches. Inflow rate for the LAD derived models are matched to the flow rate at the corresponding location in the 3D LCA model. (**G**) 2D cylindrical projection of LAD vessel from model (**F**) LAD 0. (**H**) and (**I**) Point-to-point comparison of TAESS (time-averaged endothelial shear stress) and velocity along the LAD for the 6 models. Black box marks the location of stenosis in the LAD vessel. LM – Left main, LAD – Left anterior descending, LCx – Left circumflex, Diag 1 – Diagonal 1 and OM1 – Obtuse marginal 1.

Prior studies that investigated preferential flow in side branches, coronary steal, and the influence of side branches on arterial flow provide evidence to suggest that it is crucial to include side branches in vascular geometries for accurate modeling of arterial hemodynamics^20,27,28^. To confirm the importance of side branches on determining coronary hemodynamics using CFD, we performed a systematic study of the effect of side branches using a left coronary artery model with a proximal stenosis in the left anterior descending (LAD) coronary vessel. We used an iterative process to remove side branches from the angiogram-derived left coronary artery tree model. This created a series of derivative models with fewer side branches that are more representative of coronary models used in previously published CFD studies. In addition to the complete left coronary artery model, which includes all vessels >1 mm seen on the coronary angiogram (Fig. 4A), we created a modified left coronary artery model comprised of the left main (LM), left anterior descending (LAD) and the first diagonal branch, and the left circumflex (LCX) and first obtuse marginal branch. (Fig. 4B). To further investigate the effect of the number, location and length of major and minor side branches on arterial flow we created 4 additional models of the LAD only with 3, 2, 1 or 0 side branches, corresponding to LAD 3, LAD 2, LAD 1, and LAD 0, respectively (Fig. 4C–F).

To examine time-averaged ESS (TAESS) between models with different numbers and locations of side branches, a point-to-point comparison along the length of the LAD was performed for the 6 models to compute the differences in TAESS and average velocity (Fig. 4G–I). At the site of the stenosis in the LAD where patterns of high TAESS (>2.0 Pa) followed by low TAESS (<2.0 Pa) were identified in the complete left coronary artery (black box), differences in TAESS up to 21.0 Pa were found between the 6 different models at this site thereby confirming the importance of side branches in the models. Furthermore, average TAESS in the LAD 0 model, without any side branches, was 4.75 Pa as compared to 0.25 Pa observed in model LAD 3, with 3 side branches, a difference of more than an order of magnitude (Fig. 4J). This marked difference in TAESS between the models is attributable, in part, to the overall reduction in coronary volume when accounting for blood flow into the LAD model without side branches and a sharp increase in average velocity (0.16 m/s) as compared to the full left coronary circulation. This results in significant changes in flow distribution across different branches, which highlights the critical effects of side branches on ESS and coronary hemodynamics.

### Analysis of hemodynamic risk factors in arterial models

**Figure 5:**
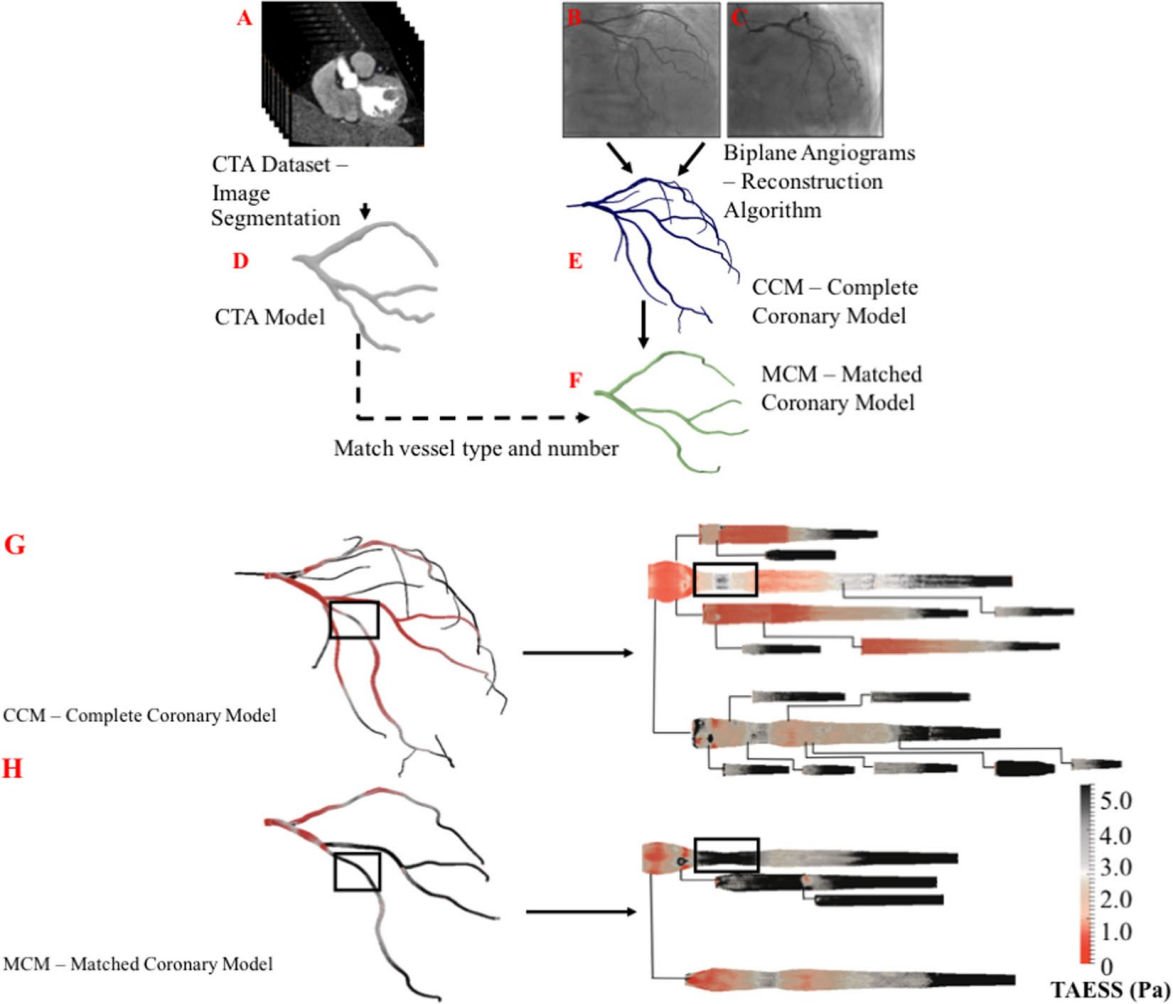
Deriving 3D hemodynamic variables from 2D angiograms. (**A**) Image stack from CTA data. (**B,C**) Patient angiograms used in the reconstruction algorithm. (**D**) CTA reconstructed 3D model. (**E**) Angiogram reconstructed 3D model – CCM. (**F**) Angiogram 3D model matched to CTA model with respect to type and number of vessels and side braches – MCM. (**G,H**) Represents 3D and 2D pulsatile endothelial shear stress maps. Box identifies the location of LAD stenosis. CCM models show lower pulsatile endothelial shear stress (<1.88 Pa) magnitude in the stenosis region than the MCM model (>4.76 Pa). Low pulsatile endothelial shear stress (<2.0 Pa) is known as pro-atherogenic indicator, this feature is adequately obtained by the CCM simulation and not the MCM simulation.

Having established that side branches have a significant influence on arterial hemodynamics, we next performed fluid simulations in the angiogram-derived complete coronary models with all vessels >1 mm (CCM) and angiogram-derived coronary models that were pruned to match the model derived from the CTA, referred to as the matched coronary model (MCM). The MCM were created to eliminate any subtle bias imposed by the different reconstruction algorithms used for the CTAs and angiograms while capturing the number, size, and length of side branches in the CTA reconstructions (Fig. 5A). To demonstrate the utility of this approach, we compared ESS in the CCM and MCM geometries at the region of a stenosis (Fig. 5G–H). Here, the ESS is higher (>5.0 Pa) in the MCM than the CCM supporting our observation that when the model geometry has fewer side branches, the ESS is overestimated (Fig. 5G–H).

We next investigated local differences in ESS in the vessel geometries by performing a point-to-point comparison between the CCM and MCM models from all patients. After excluding sections corresponding to branches in the CCM that were absent in the MCM models, a total of 17,757 sections from 15 left coronary arteries and 12 right coronary arteries remained common to both models. A direct comparison between matched sections from the CCM and MCM geometries revealed that 72.3% sections had a difference of >0.5 Pa and only 27.7% had a difference of 0.5 Pa. This suggests that differences in the number of side branches between the models can lead to inaccuracies in ESS at a local level.

**Figure 6:**
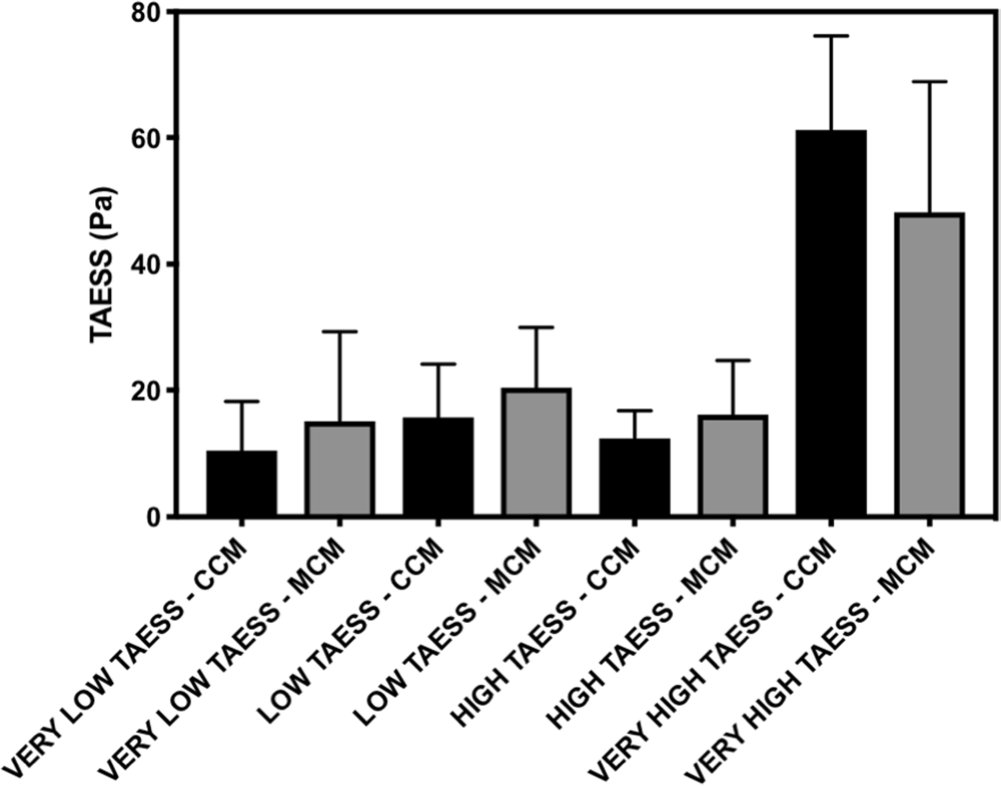
Difference in severity of endothelial shear stress (ESS) between complete and matched coronary models analyzing all vessels. Low ESS < 1.0 Pa. Intermediate 1.0 Pa < ESS < 2.0 Pa. High 2.0 Pa < ESS < 3.0 Pa. Very High ESS > 3.0 Pa. The ESS computed was time averaged over the period of the cardiac cycle. Percentages taken of 17,757 total CCM/MCM paired sections.

The relative luminal surface area distribution of ESS in paired CCM and MCM sections stratified by the range of ESS was also examined. The 17,757 paired sections were analyzed to determine the relative number of sections exposed to low (<1.0 Pa), intermediate (1.0 Pa–<2.0 Pa), high (2.0 Pa–<3.0 Pa) and very high (>3.0 Pa) ESS. There was a significant difference in the percent distribution of sections per each category of ESS for both the CCM (10.6 ± 1.5 vs. 15.8 ± 1.7 vs. 12.5 ± 1.0 vs. 61.2 ± 3.0%, p < 0.0001) and MCM (15.2 ± 2.8 vs. 20.4 ± 1.9 vs. 16.2 ± 1.7 vs. 48.2 ± 4.1%, p < 0.0001) models. The mean difference in ESS between the CCM and MCM models was significantly different across the distribution of ESS (−4.6 ± 2.6 vs. −4.7 ± 2.2 vs. −3.7 ± 1.8 vs.13.0 ± 4.4. %, p < 0.006). These differences suggest that low and intermediate ESS is overestimated whereas very high ESS is underestimated in the MCM models (Fig. 6). We also investigated differences in ESS on a per vessel basis for the LAD, LCX and RCA in both the CCM and MCM models. There was a significant difference in the percentage of sections per vessel exposed to very low ESS for both the CCM (p < 0.05) and the MCM models (p = 0.01); however, there were no differences between the vessels exposed to low, high, or very high ESS (Supplemental Fig. 1).

**Figure 7:**
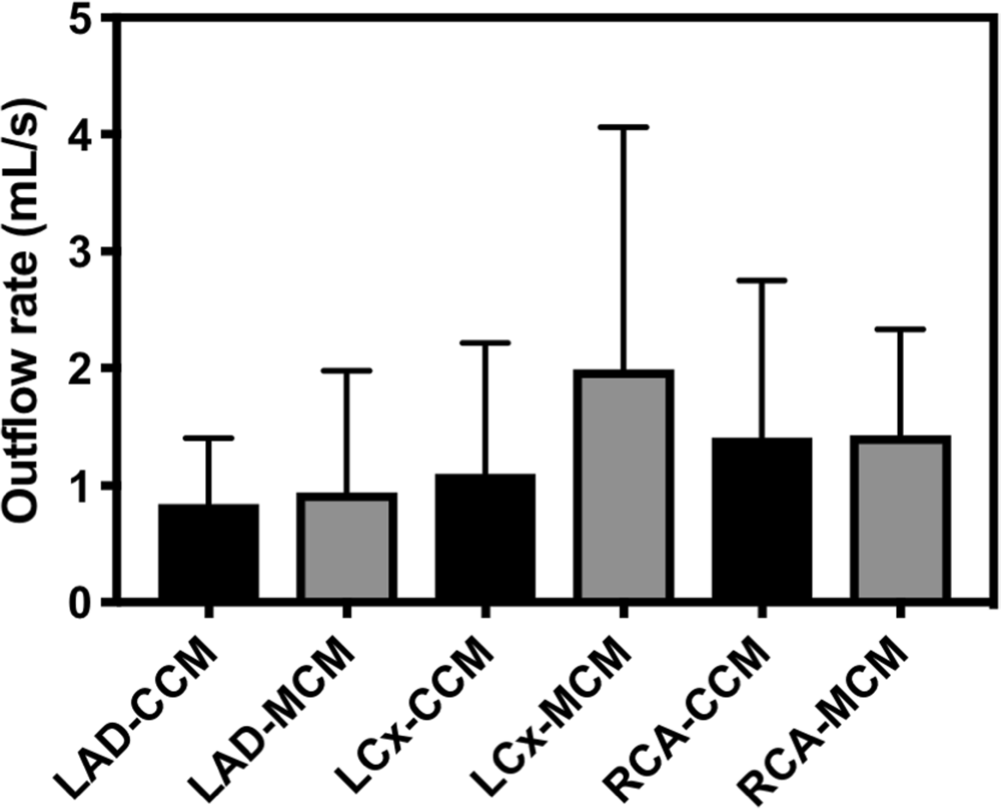
Difference in volumetric outflow between complete and matched coronary models in major vessel.

Mean volumetric outflow rates were also computed for the CCM and MCM models in the LAD, LCx and RCA vessels (Fig. 7). Identical pulsatile waveforms were applied to CCM and MCM models with the mean velocity of 28 cm/s averaged over one cardiac cycle. As anticipated the outflow rates in the CCMs (3.32 ml/s) were lower than the MCM models (4.41 ml/s) due to the greater number of side branches. The LCX had a significantly higher mean flow rate in the MCMs (2.0 0.5 ml/s) than in the CCMs (1.1 0.3 ml/s, p < 0.05) while there were no significant differences between models in the LAD and RCA. These results overall suggest that without comprehensive anatomic detail, such as inclusion of smaller side branches, hemodynamic risk-factors considerably vary.

## Discussion

The main findings of this study are: (1) 3D hemodynamic risk factors such as ESS and volumetric outflow can be derived directly from conventional angiography data; (2) anatomically precise and validated arterial tree reconstructions that include all identifiable side branches >1.0 mm are critical to conduct higher fidelity biomechanical studies; and (3) patient-specific simulations that are based on arterial trees with only a subset of or no side branch vessels, overestimate ESS and volumetric outflow in arterial vessels. Our findings are in line with prior reports demonstrating that side branches had an effect on ESS^20,27,29^. Wellnhofer *et al*. evaluated the effect of side branches in 17 reconstructed RCAs, including models obtained from 7 patients without coronary disease, 5 from patients with disease and 5 from patients with aneurysmal dilation of the vessel. They reported a reduction of 78.7% in coronary volume flow and differences in ESS up to 12 Pa in the model without side branches as compared to the complete model^28^. In the current work, we performed a systematic removal of side branches from the LAD and found a similar reduction in coronary volume flow of 72% (1.9 ml vs. 0.54 ml). We also found a maximum difference in regional ESS of 11.0 Pa between the complete LCA model with all vessels and LAD without any side branches. By performing analyses in 17 LCA models (in addition to 12 RCA models), where a greater influence of side branches is expected relative to the RCA due to the branching patterns and side branches associated with the LAD and LCx, we now demonstrate that the relationship between side branches, coronary volume flow, and ESS holds for left coronary vessels as well. All hemodynamic simulations performed in this study are transient with pulsatile ESS calculation averaged over one cardiac cycle and are thus extensible to compute ESS gradients and oscillatory shear index. While the flow separation is determined by vessel diameters, CFD in this study takes into account flow through each vascular branch based on the total vessel volume and physiological resistance at each vessel outlet. Total coronary flow under resting conditions can be derived from myocardial mass, given that $Q\propto {M}_{myo}^{b}$^18,30^. Myocardial mass in turn can be estimated from CTA images and the inclusion of myocardial mass may compensate for fewer side branches^18,31^. However, the CA-based CFD method proposed in this study directly uses the resting coronary flow volume to determine the inlet boundary conditions and does not depend on the myocardial mass calculation.

Other studies have also investigated the influence of side branches on ESS. One study examined 21 coronary geometries reconstructed from a novel intravascular optical coherence tomography (OCT)- 3D angiography fusion method^20^. Here, the average ESS was found to be 4.64 Pa lower in models that incorporated side branches (tree models) compared to the models with no side branches (single conduit models). Our study also found that ESS results for CCMs, which include all side branches >1.0 mm, were 2.79 Pa lower than the MCMs, which had fewer and/or truncated side branches. While the prior study evaluated models including the main vessels (LAD, LCx and RCA) with and without side branches it did not compare the complete arterial circulation for the full tree with all vessels (such as in CCMs) with models that have only a subset of all side branches (MCMs matched to conventional CTA derived models). These differences have led investigators to conclude that excluding side branches from arterial models significantly decreases the ability to detect regional pathological ESS in the vessels^29^.

In contrast to prior studies, the geometric reconstructions used in our study are solely obtained from traditional routine 2D angiography data, the gold standard and most commonly used anatomic test to assess coronary artery disease^12,13,32^. Current CTA-based CFD methods can derive local hemodynamic variables and diagnostic metrics such as fractional flow reserve (FFR); however, among patients who undergo percutaneous coronary interventions only a limited patient population undergoes CTA, whereas they all undergo CA^13,17^. Several studies that have introduced CA-based CFD methods analyze only vulnerable plaques or focal regions of the coronary tree^32–35^. The regions corresponding to smaller or partially overlapped vessels or the full coronary arterial tree are often not fully incorporated in the 3D reconstruction. Further, the vessel geometry (e.g., tortuousity) and cross-sectional diameters (e.g., stenotic lesion) in the 3D model are difficult to correlate with image data used for reconstruction^27,36^. This study addresses these limitations by reconstructing complex left and right coronary arterial trees with all identifiable vessels from the pair of biplane angiograms. Hemodynamic analysis based on CA data used in this study can, thus, significantly increase the target patient population that are candidates for ESS and other hemodynamic risk factor analysis without requiring additional imaging modalities to be employed in order to obtain 3D geometries that include all side branches >1 mm. A recent study also used routine CA imaging data to reconstruct coronary arterial geometry; however, the study models blood flow using a lumped parameter model treating the arterial network as electrical circuit and vessel segments as resistors instead of relying on traditional CFD equations^37^. While reduced order flow models such as lumped models and 1-dimensional models can be used to derive macroscopic hemodynamic variables such as pressure gradient and flow changes, they are not feasible to compute local hemodynamic quantities such as ESS and velocity profiles^38,39^. Our CA-based CFD method can derive local pressure fields and can naturally extend itself to calculate diagnostic metrics such as FFR. Therefore, this study constitutes a first step towards the development of computational strategies which would allow for an accurate study of patient specific hemodynamic profiles from conventional 2D coronary angiography imaging. The availability of such strategies holds promise for enabling important advances in the biomechanical understanding of coronary artery disease.

One limitation of this study is that we validate the geometric reconstruction of angiogram models by comparing them to CTA-derived models; however, other imaging modalities, such as intravascular ultrasound and optical coherence tomography, may provide superior resolution for validating our geometries. While these intravascular imaging studies were not available in the present study, the close agreement between angiography of the 3D phantom and the patient’s angiogram provides an additional level of support to show that our 3D geometries accurately captured the level of resolution available from routine coronary angiograms. For the hemodynamic simulations, we assume blood to be a Newtonian fluid and have rigid walls for modeling blood vessels. These are valid assumptions often made in CFD studies due to the shear rates found in vessels of the size of the coronaries^18,28^. Furthermore, in our previous work we have reported successful validation of our CFD modeling tool – HARVEY – with *in vitro* experiments using particle image velocimetry in a geometry of an aortic coarctation. As the flow regimes in the coronary artery are not different from the aorta, we believe the findings from the validation study in the aorta to be consistent for coronary artery modeling^40^. In addition to ESS, temporal and spatial gradients of ESS and oscillatory shear index (OSI) have been suggested as important hemodynamic risk indicators by previous work^19,36^. This study, however, already incorporates transient simulations; thus, we can easily compute ESS gradients and OSI. Studies such as the one by Peiffer *et al*. have indicated that point-by-point comparison for relating ESS and atherosclerosis is challenging due to difficulties in regional matching^41^. This study addresses the region-mismatch limitation by first aligning the centerlines of the MCM and CCM models and compute centerpoints at identical resolutions for both sets of geometries. Thus, having closely aligned centerlines and centerpoints locations we were able to conduct point-to-point comparison and minimize the error due to geometric misalignment.

There is increasing recognition that the hemodynamic evaluation of coronary arteries and associated disease pathology improves patient outcomes. Thus, a pipeline to go from conventional angiography data to deriving potential hemodynamic risk factors from 3D simulations using geometries that include all side branches for a high degree of precision has the potential to provide a simple, elegant and cost-effective solution to improve diagnosis and treatment planning for patients with atherosclerosis. The complete coronary trees that take into account flow through all side branches are a cornerstone of the pipeline and are required for accurate computation of ESS and pressure gradients. By contrast, models that have only a subset of side branches are inadequate for biomechanical studies as they overestimate the volumetric outflow and ESS and lead to inaccuracies in hemodynamic risk associated with these factors.

## Supplementary information

Supplementary Material
