# Supplementary material for: The importance of side branches in modeling 3D hemodynamics from angiograms for patients with coronary artery disease
Source: Sci Rep. 2019 Jun 20;9:8854. doi: 10.1038/s41598-019-45342-5 (PMC6586809; doi:10.1038/s41598-019-45342-5)
Supplement: Supplementary file 1 — Supplementary Material [file 41598_2019_45342_MOESM1_ESM.pdf]

The importance of side branches in modeling 3D hemodynamics from angiograms for patients with coronary artery disease

Madhurima Vardhan[1], John Gounley[1], S. James Chen[2], Andrew M. Kahn[3], Jane A. Leopold[4], and Amanda Randles[1,*]

[1]Department of Biomedical Engineering, Duke University, Durham, 27705, USA. [2]Department of Medicine/Cardiology, University of Colorado AMC, 80045, USA. [3]Division of Cardiovascular Medicine, University of California San Diego, 92103, USA. [4]Division of Cardiovascular Medicine, Brigham and Women's Hospital, 02115, USA.

[*] amanda.randles@duke.edu

Supplementary Material

FIGURES

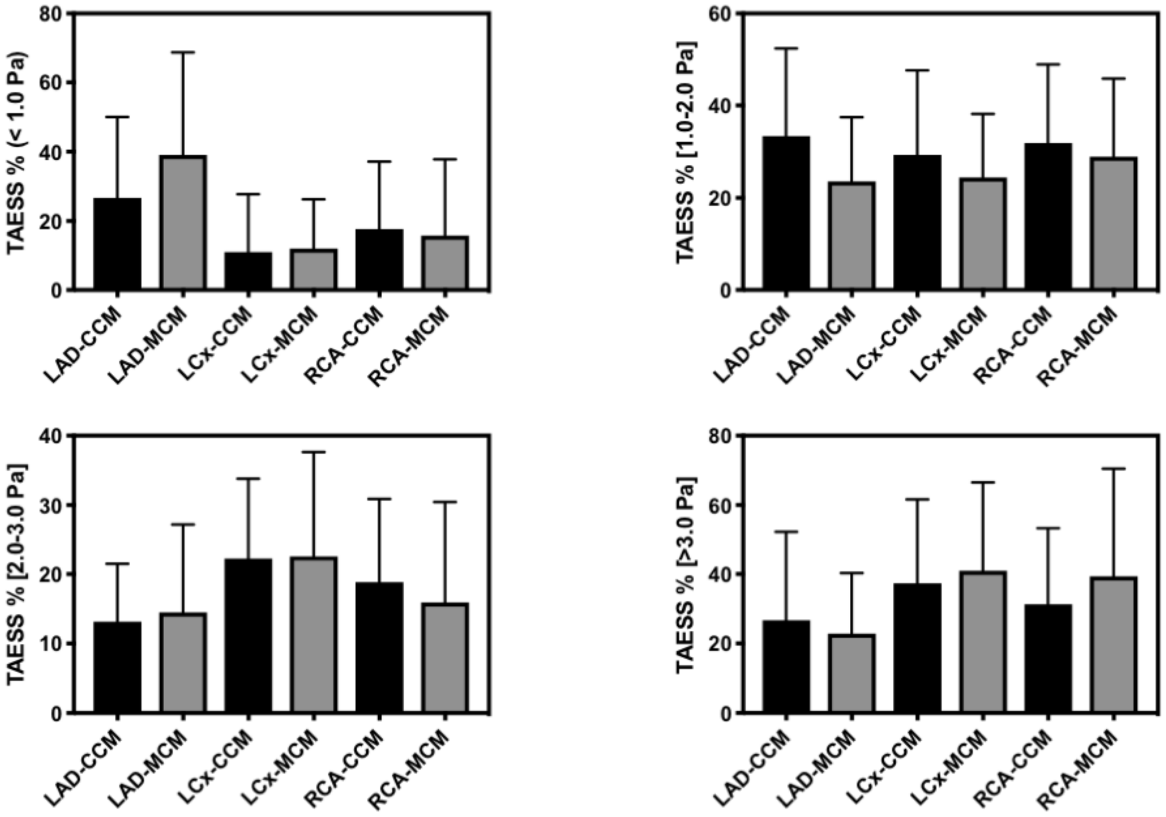

Supp. Figure 1: Difference in ESS between complete and matched coronary models depending on severity of endothelial shear stress (ESS) in major vessels. Low ESS < 1.0 Pa. Intermediate 1.0 Pa < ESS < 2.0 Pa. High 2.0 Pa < ESS < 3.0 Pa. Very High ESS > 3.0 Pa. The ESS computed was time averaged over the period of the cardiac cycle. Percentages taken of 17,757 total CCM/MCM paired sections.

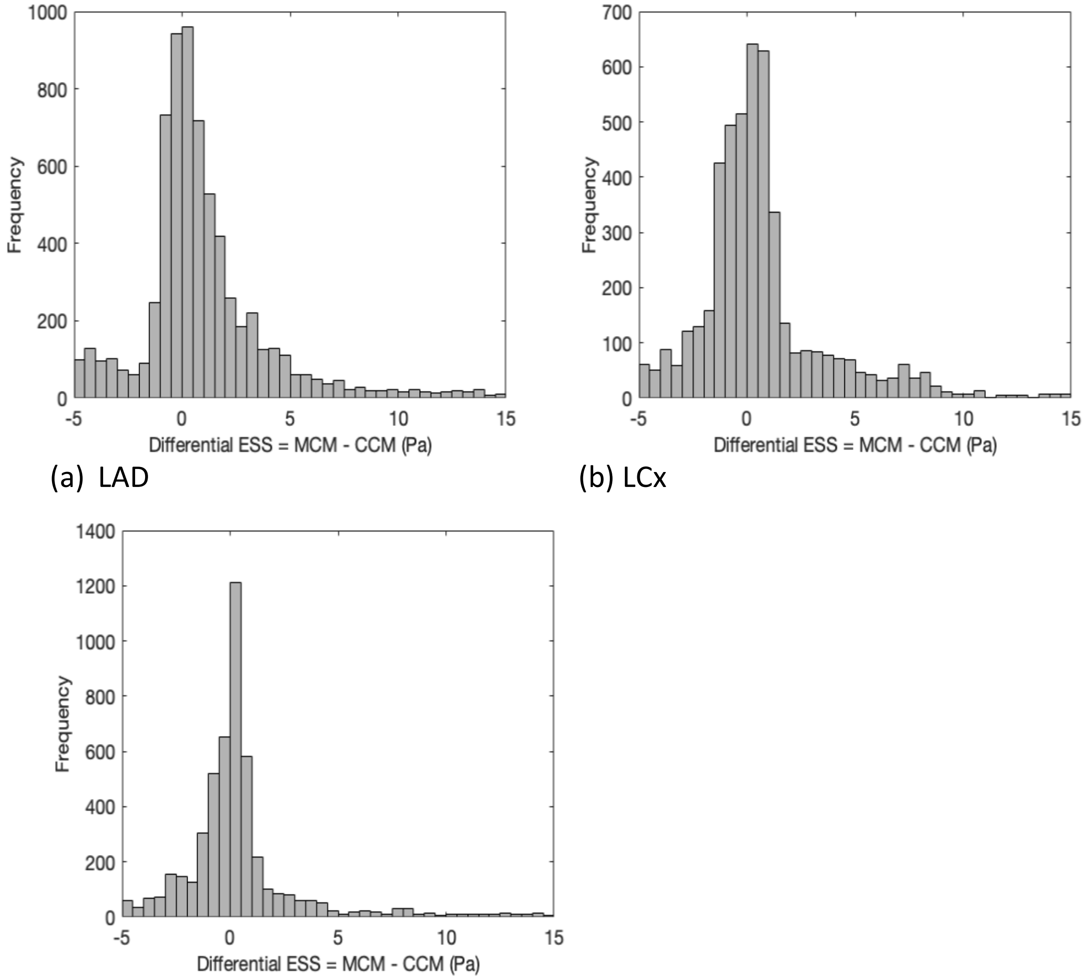

(a) LAD

(b) LCx

(c) RCA

Supp. Figure 2: Difference in time averaged endothelial shear stress at corresponding points along the (a) Left anterior descending LAD (b) Left circumflex LCx (c) Right coronary artery RCA.

TABLES

Supp. Table 1: Outflow Left Anterior Descending (LAD) and Left Circumflex (LCx)

Case	LAD-CCM (mL/s)	LAD-MCM (mL/s)	LCx-CCM (mL/s)	LCx-MCM (mL/s)
1	1.77	0.53	2.87	0.66
2	0.52	0.56	0.49	0.68
3	0.87	0.99	1.16	2.10
4	0.88	0.23	1.30	1.48
5	1.17	0.44	1.15	8.89
6	1.72	0.47	0.37	1.98
7	0.83	1.01	4.17	3.04
8	1.77	0.58	0.00	1.09
9	0.69	0.90	1.20	3.05
10	0.67	1.33	0.60	1.39
11	0.21	0.75	0.54	0.85
12	0.95	1.50	1.63	2.09
13	0.16	0.00	0.31	1.24
14	0.09	0.47	0.17	0.71
15	0.35	4.39	0.54	0.59

Supp. Table 2: Outflow Right Coronary Artery

Case	RCA-CCM (mL/s)	RCA-MCM (mL/s)
1	0.94	1.40
2	0.44	1.82
3	0.75	2.01
4	1.08	0.82
5	4.26	2.42
6	0.56	0.27
7	3.22	2.48
8	0.54	0.50
9	2.01	2.32
10	0.31	0.26
